# Analysis of Combination Drug Therapy to Develop Regimens with Shortened Duration of Treatment for Tuberculosis

**DOI:** 10.1371/journal.pone.0101311

**Published:** 2014-07-08

**Authors:** George L. Drusano, Michael Neely, Michael Van Guilder, Alan Schumitzky, David Brown, Steven Fikes, Charles Peloquin, Arnold Louie

**Affiliations:** 1 Institute for Therapeutic Innovation, College of Medicine, University of Florida, Lake Nona, Florida, United States of America; 2 Laboratory of Applied Pharmacokinetics, School of Medicine, University of Southern California, Los Angeles, California, United States of America; 3 Infectious Diseases PK Laboratory, College of Pharmacy, University of Florida, Gainesville, Florida, United States of America; Cambridge University, United Kingdom

## Abstract

**Rationale:**

Tuberculosis remains a worldwide problem, particularly with the advent of multi-drug resistance. Shortening therapy duration for *Mycobacterium tuberculosis* is a major goal, requiring generation of optimal kill rate and resistance-suppression. Combination therapy is required to attain the goal of shorter therapy.

**Objectives:**

Our objective was to identify a method for identifying optimal combination chemotherapy. We developed a mathematical model for attaining this end. This is accomplished by identifying drug effect interaction (synergy, additivity, antagonism) for susceptible organisms and subpopulations resistant to each drug in the combination.

**Methods:**

We studied the combination of linezolid plus rifampin in our hollow fiber infection model. We generated a fully parametric drug effect interaction mathematical model. The results were subjected to Monte Carlo simulation to extend the findings to a population of patients by accounting for between-patient variability in drug pharmacokinetics.

**Results:**

All monotherapy allowed emergence of resistance over the first two weeks of the experiment. In combination, the interaction was additive for each population (susceptible and resistant). For a 600 mg/600 mg daily regimen of linezolid plus rifampin, we demonstrated that >50% of simulated subjects had eradicated the susceptible population by day 27 with the remaining organisms resistant to one or the other drug. Only 4% of patients had complete organism eradication by experiment end.

**Discussion:**

These data strongly suggest that in order to achieve the goal of shortening therapy, the original regimen may need to be changed at one month to a regimen of two completely new agents with resistance mechanisms independent of the initial regimen. This hypothesis which arose from the analysis is immediately testable in a clinical trial.

## Introduction

*Mycobacterium tuberculosis* (Mtb) infects one-third of the world's population. The World Health Organization estimates that in 2011 there were 8.7 million new cases and 1.4 million deaths caused by this microbe [1]. Standard treatment for drug susceptible Mtb consists of two months of rifampin, isoniazid, pyrazinamide and ethambutol during the intensive phase of therapy followed by four months of rifampin and isoniazid during the continuation phase [2]. Treatment failure caused by initially drug-susceptible (DS) Mtb is sometimes due to emergence of antibiotic-resistant isolates. The major gap for optimal TB therapy is the absence of an effective short (circa 2 months) regimen that is active against both DS- and Multi-Drug Resistant (MDR)-TB. The ability to obtain maximal rates of kill of Mtb while suppressing resistance emergence is our best hope of markedly shortening duration of therapy.

Recently a number of anti-TB drugs have entered clinical trials (e.g. bedaquiline, delamanid, PA-824, SQ109, sutezolid) or are approved for the treatment of other infections (clofazimine) [3], [4]–[6]. A unifying theme shared by these drugs is that their unique mechanisms of action do not confer cross resistance to current first and second line drugs.

Combination therapy is a proven method for suppressing resistance emergence in patients with active Mtb infection [7]. Choosing the right drugs in combination is critical to achieving the goal of rapid kill with resistance suppression. Isoniazid is antagonistic with PZA and perhaps rifampin in the standard regimen in a murine evaluation [8]. Our laboratory has demonstrated in the Hollow Fiber Infection Model (HFIM) that moxifloxacin and rifampin are antagonistic for bacterial cell kill but do provide good suppression of amplification of resistant sub-populations; the antagonism has been validated in the murine aerosol challenge model [9], [10].

We need to develop a methodology for rationally identifying optimal combinations for clinical trial. Our HFIM has evaluated a large number of anti-TB agents, both alone and in combination [11]–[16] with the results correlating with both murine data as well as clinical data.

In this evaluation we examined linezolid and rifampin, alone and in combination against Log-phase *M. tuberculosis*. Rifampin was chosen because it is one of our best agents against both Log-phase organisms as well as organisms in Non-Replicative Persister phase [9]. Linezolid has shown promising activity as a single agent in patients with XDR TB [17].

Greco and colleagues developed the Universal Response Surface Approach (URSA) in the oncology realm [18] as a mathematically rigorous approach to determining the interaction of drugs (synergy, additivity, antagonism). We have extended this approach by also considering *a priori* drug-resistant subpopulations.

Because the approach is fully parametric, the results can be submitted for Monte Carlo simulation. In so doing, we can identify drug doses that will 1) obtain maximal bacterial cell kill and 2) suppress resistant subpopulation amplification for both drugs and do so for a population of simulated patients. This will provide a rational way forward to choose optimal combinations that will lead to shortened therapy durations.

## Results

### MIC determination and mutation frequency

The H37Rv strain used for these experiments had an MIC to linezolid of 1.0 mg/L and 0.25 mg/L to rifampin. The mutational frequency to resistance for linezolid was −6.93±0.44 Log_10_ (CFU) at 2 mg/L (2xMIC); for rifampin, it was −5.77±0.48 Log_10_(CFU) at the critical concentration of 1 mg/L.

### HFIM evaluation of linezolid and rifampin alone and in combination

**Figure 1:**
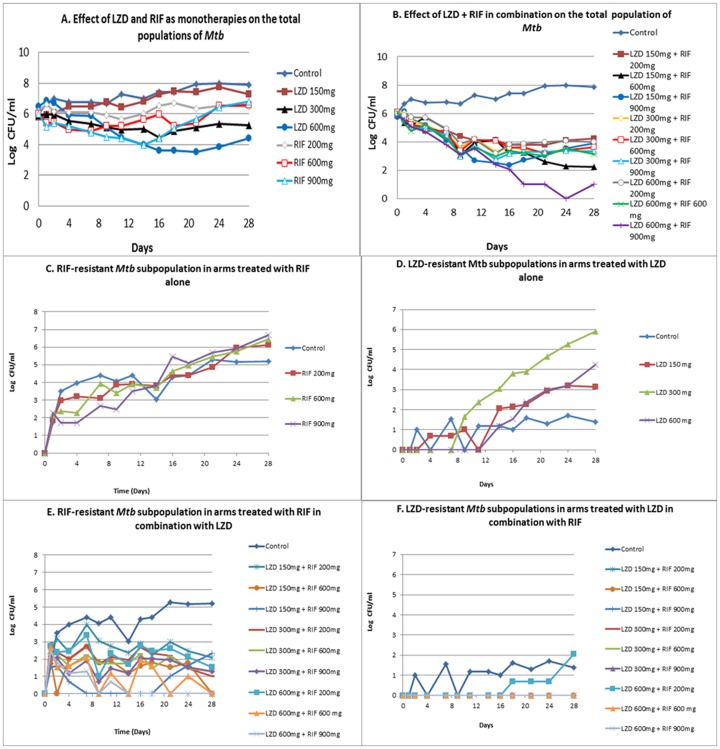
Effect of Linezolid (LZD) or Rifampin (RIF) alone and in combination on the total colony counts of *Mycobacterium tuberculosis* (Mtb) (Panels A and B) and on the less susceptible subpopulations (Panels C–F) as determined in a Hollow Fiber Infection Model.

Non-protein-bound drug exposures (Area Under the concentration-time Curve–AUC) of rifampin consistent with doses of 200, 600 and 900 mg daily and of linezolid at exposures of 150, 300 and 600 mg daily were examined. These agents were also examined in combination in all possible two-drug regimens (9 regimens). A no-treatment-control was also included. The single agent regimens (including control) total colony counts are displayed in Figure 1 Panel A. No single agent regimen produced any substantial change in total colony counts over the 28 days of observation. In addition, samples were also quantitatively cultured on plates infused with twice the MIC of linezolid (2×1 mg/L) or with the rifampin critical concentration of 1 mg/L (4×MIC). All single agent regimens allowed emergence of resistance (Figure 1, Panel C, D). Resistant isolates were recovered after 7–10 days.

Combination therapy regimens effect on the total Mtb population is shown in Figure 1, Panel B. Surprisingly, most combination regimens also allowed resistance emergence (Figure 1, Panels E, F). These regimens, did, however result in a multi-Log decline in total bacterial population in some instances. Only regimens with 600 mg or 900 mg of rifampin daily in combination with 600 mg daily of linezolid had no organisms recoverable on resistance plates by experiment end.

### Mathematical population model for all regimens

In this model, we examined the concentration-time curves of each agent either alone or in combination. These concentration-time profiles were analyzed by the first two differential equations (see Methods for a full model description). The third differential equation described the impact of drug exposure on the total bacterial population, which included the population fully susceptible to both agents, the subpopulation resistant to rifampin, but sensitive to linezolid and the subpopulation resistant to linezolid, but susceptible to rifampin. The fourth and fifth differential equations described the impact of combination therapy on subpopulations resistant to drug 1/sensitive to drug 2 and resistant to drug 2/sensitive to drug 1. No organisms resistant to both drugs were recovered at baseline.

The fit of the model to the data was acceptable, as seen below:

1. Observed-Predicted Regression for All Linezolid Concentrations 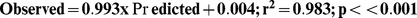

2. Observed-Predicted Regression for All Rifampin Concentration 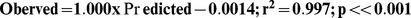

3. Observed-Predicted Regression for All Total Colony Counts 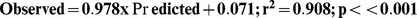

4. Observed-Predicted Regression for All Colony Counts (Linezolid-Resistant) 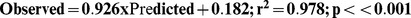

5. Observed-Predicted Regression for All Colony Counts (Rifampin-Resistant) 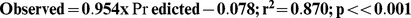

These regressions represent the observed-predicted plots using the Bayesian-posterior estimates. The median parameter vector was employed to obtain the Bayesian estimates for each system output. The point estimates of all the mean and median parameter values and standard deviations are displayed in Table 1.

**Table 1:** Parameter Values From a Combination Chemotherapy Mathematical Model.

Parameter	Units	Mean	Median	S.D.
**V_1_**	**L**	**83.5**	**81.9**	**22.4**
**CL_1_**	**L/h**	**6.20**	**6.19**	**0.748**
**V_2_**	**L**	**139**	**141**	**10.9**
**CL_2_**	**L/h**	**30.8**	**31.5**	**1.77**
**POPMAX**	**CFU/ml**	**7.85×10^9^**	**7.24×10^8^**	**1.75×10^10^**
**K_gs_**	**h^−1^**	**0.100**	**0.107**	**0.0464**
**K_ks_**	**h^−1^**	**0.235**	**0.170**	**0.130**
**E_501s_**	**mg/L**	**0.527**	**0.358**	**0.389**
**E_502s_**	**mg/L**	**2.72**	**2.33**	**2.38**
**α_s_**	**-----**	**−0.954**	**−0.232**	**4.73**
**K_gr1_**	**h^−1^**	**0.0232**	**0.0198**	**0.0116**
**K_kr1_**	**h^−1^**	**0.274**	**0.318**	**0.113**
**E_50_1r1_**	**mg/L**	**13.5**	**13.8**	**2.49**
**α_r1_**	**-----**	**−4.55**	**−6.11**	**3.39**
**K_gr2_**	**h^−1^**	**0.127**	**0.133**	**0.0757**
**K_kr2_**	**h^−1^**	**0.251**	**0.190**	**0.112**
**E_50_2r2_**	**mg/L**	**5.92**	**6.01**	**0.829**
**α_r2_**	**-----**	**−0.431**	**−0.950**	**2.48**
**H_1s_**	**-----**	**4.60**	**4.64**	**1.85**
**H_2s_**	**-----**	**2.26**	**2.35**	**1.07**
**H_1r1_**	**-----**	**18.4**	**20.3**	**6.73**
**H_2r2_**	**-----**	**15.3**	**15.8**	**3.23**
**INIT_4_**	**CFU/ml**	**1.88**	**2.72**	**1.87**
**INIT_5_**	**CFU/ml**	**1.50**	**1.98**	**1.14**

**S.D. =  Standard Deviation.**

The value of the interaction parameter “α” for each of the populations determines the drug interaction (synergy, additivity, antagonism) for the combination regimens. The value of these parameters and attendant 95% confidence interval is displayed in Table 2.

**Table 2:** Interaction parameters (α) for fully susceptible (S), resistant to linezolid (L), and resistant to rifampin (R) organism populations and 95% confidence intervals.

	α _Susceptible_	α _L-resistant_	α _R-resistant_
**Mean**	**−0.709**	**−4.347**	**−0.436**
**Median**	**−0.232**	**−5.725**	**−0.917**
**95% CI**	**−8.701–7.295**	**−11.309–2.615**	**−4.85–4.422**

As can be seen, all α values are negative. However, for each, the 95% confidence interval overlaps zero, meaning the combination tends toward antagonism (the negative α-value), but is not significant and would be accorded a definition of additivity.

**Figure 2:**
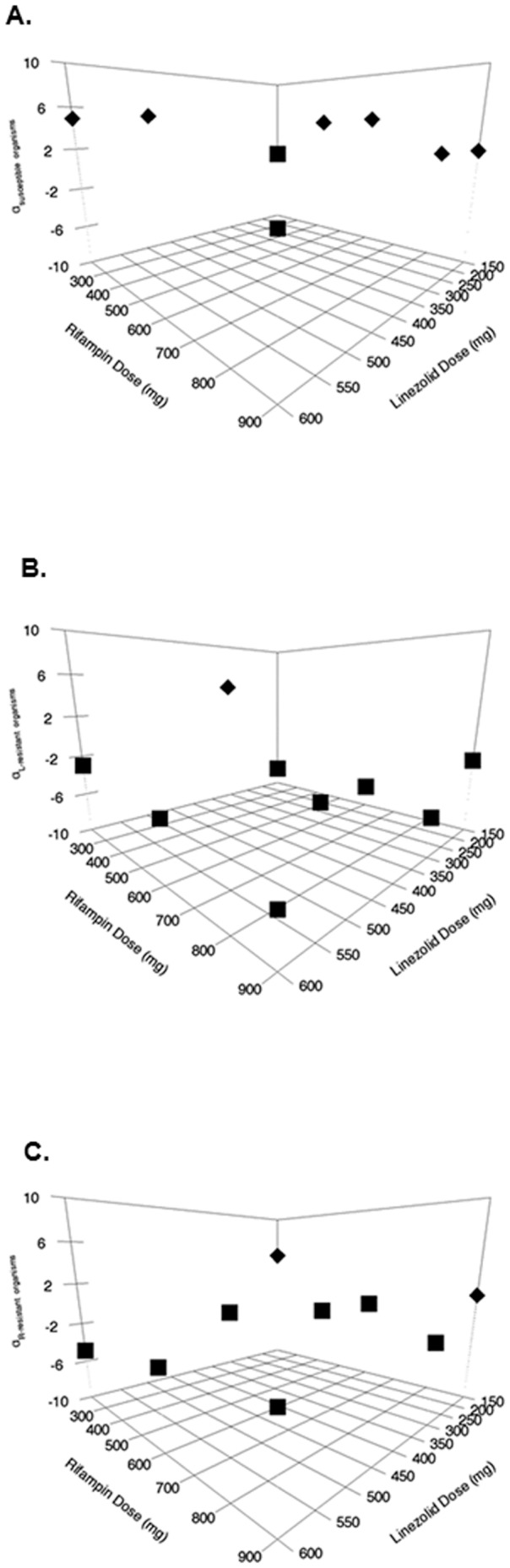
Plots of the α-values (an index of drug interaction for effect) for different combination regimens of linezolid plus rifampin for A. Fully-Susceptible Organisms; B. Linezolid-Resistant Organisms; C. Rifampin-Resistant Organisms. Diamonds indicate positive α-values; Squares indicates negative α-values.

Of equal or greater importance, we can obtain Bayesian estimates of the interaction parameters for each of the 9 combination therapy regimens. These values of α are displayed by regimen in Figure 2 panels A–C. It is apparent by inspection that some values of α are positive (tending to synergy), but that these positive values are distributed in different parts of the space for each population/subpopulation, meaning that identifying a regimen optimal for each of these populations/subpopulations is not straightforward.

### Monte Carlo simulation

**T**he greatest decrement of total bacterial population while maintaining suppression of amplification of resistant subpopulations is only attained by combination regimens of linezolid 600 mg daily in combination with either 600 or 900 mg of rifampin daily (Figure 1). The exposure targets for these simulated regimens are AUC/MIC ratios of 98.9 of linezolid plus 84.6 of rifampin for the first regimen and 98.9 of linezolid and 123.2 for rifampin for the second regimen.

We generated a 1000-subject Monte Carlo simulation for Area Under the concentration-time Curve (AUC) for the combination of 600 mg/600 mg of linezolid/rifampin. We employed previous determinations of rifampin and linezolid penetration into the Epithelial Lining Fluid (ELF) [19]–[21]. The values employed were for total ELF values, as we are unaware of any information regarding free fraction in ELF. The percent penetration (AUC_ELF_/AUC_Plasma_ Ratio) was employed to calculate the distributions of AUC_ELF_ for linezolid. To obtain this distribution, we were kindly provided with the data from the publication of McGee et al [22]. The subset of the patients (infected with *M. tuberculosis*) who received linezolid 600 mg daily were subjected to population pharmacokinetic modeling with NPAG. The first distribution calculated was for 1,000 simulated patients for AUC_Plasma_. This distribution was then multiplied by percent penetration into ELF. We employed the average penetration of the calculated penetration of the studies of Honeybourne et al [19] and Boselli et al [20]. For rifampin, the analysis of Goutelle et al [21] provided an explicit parameter vector that allowed direct calculation of the AUC_ELF_ for a 600 mg dose.

We then determined the frequency with which these regimens would achieve the AUC/MIC targets for linezolid/rifampin of 98.9/123.2 as measured in the HFIM. To ultimately obtain an expectation for target attainment we employed the MIC distribution for linezolid from the paper of Ahmed et al [23] and for rifampin from the paper of van Klingeren et al [24]. For a regimen of linezolid 600 mg plus rifampin 600 mg, the exposure which suppressed resistance amplification, but which did not achieve the maximal cell kill the AUC/MIC was attained (expectation over the MIC distribution) 96% of the time for linezolid and 60% of the time for rifampin. Assuming orthogonality of probabilities, both exposures would be attained 57.6% of the time.

For the linezolid 600 mg daily plus rifampin 900 mg daily regimen, we employed the data from a pharmacokinetic evaluation of higher rifampin doses in TB-infected patients by Boeree et al [25]. The abstract did not provide measures of variability, but did provide point estimates of the mean AUC for doses of 10, 20, 25 and 30 mg/kg per day, as determined on day 7 of dosing. We used the ratio of AUC's of 10 mg/kg/day to 20, 25 and 30 mg/kg/day, which were 4.28, 5.11 and 7.20, respectively. These increases in AUC_Plasma_ were employed as multipliers for the AUC_ELF_ as determined above from the data of Goutelle et al [21].

For the 20 mg/kg/day dose, the rifampin expected target attainment was 88.0% and the combined regimen expected target attainment was 84.5%. For the 25 mg/kg/day dose, these values were 91.4% for rifampin and 87.7% for the combination. For the 30 mg/kg/day rifampin dose, these results were 96.9% for rifampin and 93.0% for the combination.

**Figure 3:**
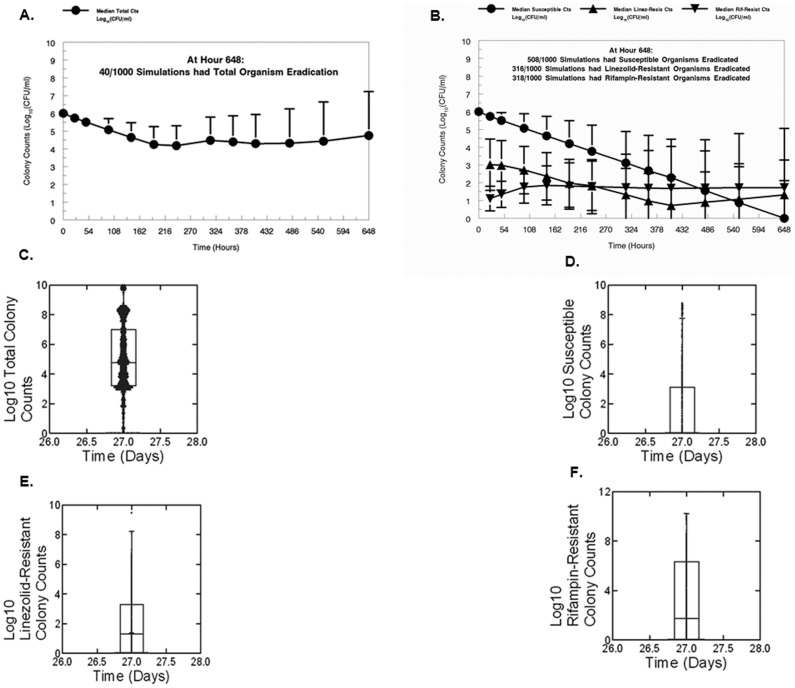
System simulation (1,000 iterate Monte Carlo simulation) for total colony counts (A), susceptible counts and subpopulations less-susceptible to the study drugs (LZD and RIF) from the Bayesian posterior parameter vectors (B). In Panels A and B, the median values and the standard deviations are displayed. In Panels C–F, the box and whisker plots (median-line; 25^th^ and 75^th^ percentiles at the bottom and top of the box; 95^th^ percentile is displayed at the top of the figure) show the distribution of colony counts for the total population (Panel C), the susceptible population (Panel D) and the less-susceptible populations for LZD (Panel E) and RIF (Panel F) simulated at the last day of the experiment.

We also wished to show the utility of the fully parametric modeling approach and performed a 1,000 subject simulation from the full model. In Figure 3, we show the median ± standard deviation colony counts from the simulation for a regimen of linezolid 600 mg daily plus rifampin 600 mg daily. The regimen impact upon the total bacterial population, as well as the subpopulations fully susceptible to both drugs and those resistant to either linezolid or rifampin are shown. With time, all standard deviations show an increase, because any fixed-dose regimen will generate a broad range of exposures for each drug and, consequently, for both drugs in the combination. Some will be large exposures and suppress resistance, while others will be in the range optimal to amplify resistant subpopulations for one or the other drug or both.

In the total population, the regimen produces a decline just below 2 Log_10_(CFU/ml), which then regrows slightly because of resistant subpopulation amplification. Both resistant populations decline (at the median), but amplify back up with time (minimum counts for linezolid and rifampin of 0.892 and 1.585 Log_10_(CFU/ml), with hour 648 counts of 1.321 and 1.650 Log_10_(CFU/ml), respectively.

Examination of the impact of the combination chemotherapy on the fully susceptible population is most important (Figure 3B). Here, we see first order decline. By the end of the experiment slightly greater than 50% of simulated subjects had extinguished this population. Given the responses of the resistant subpopulations, we are, in essence, trading fully susceptible organisms for their resistant subpopulations which some sub-optimal exposures are amplifying.

Among the 1000 iterates, 40/1000 (4.0%) had total population eradication by day 27 (Hour 648), while 316/1000 and 318/1000 had eradication of linezolid-resistant or rifampin-resistant populations, respectively.

## Discussion

Currently, no rational way of identifying optimal combinations of agents is available. In this work we have set forth a mathematical model to allow evaluation of combination regimens both for cell kill and suppression of resistance. Because the model is fully parametric, Monte Carlo simulation can be performed to inform us about the behavior of the regimen for a population of patients. This allows explicit translation of the mathematical results to the clinic, demonstrating the impact of true between-patient variability in pharmacokinetics of both agents on the ability of the regimen to kill organisms and suppress emergence of resistance.

There are two major issues with regard to shortening therapy duration. The first is to identify a regimen that will kill organisms at as high a rate as possible. Such a regimen will then have the shortest time to an extinction event. It is also important that a regimen be robust for resistance suppression. Having multiple agents does not guarantee resistance suppression. Examination of Figure 1 shows that several combination regimens demonstrated excellent early rates of kill only to have resistance emergence ultimately occur. In order to reach the goal of shortening regimen duration, both aims need to be attained.

In this set of studies, we used our hollow fiber infection model to examine the impact of rifampin and linezolid, alone and in combination against Log-phase Mtb. For the single agent evaluations, there were exposure-responses demonstrated early on. However, in all single agent evaluations, this early exposure-response was lost due to the amplification of a less-susceptible population of organisms (Figure 1, Panels C, D).

There were nine combination therapy regimens (all possible combinations of three exposures for each agent). Surprisingly, only two of these combination regimens provided optimal resistance suppression. Only one fulfilled the task of reaching a very low total bacterial burden in addition to suppressing resistance.

Linezolid and rifampin interact in an additive way with a non-significant tendency to antagonism for kill of the WT population. The “α” value for interaction for the WT population was 4.935 for the 600 mg/600 mg regimen and was −0.265 for the 600 mg/900 mg regimen. For the resistant subpopulations, all “α” values were substantially more negative and ranged from −2.45 to −6.245. As can be seen in Figure 1, a resistant subpopulation amplified for linezolid in only 1 of 9 combination regimens (linezolid 600 mg daily/rifampin 200 mg daily). The 600 mg linezolid dose provides substantial selective pressure and the rifampin dose of 200 mg daily is not high on the exposure-response curve, allowing linezolid resistant isolates to amplify. The “α” value of −2.995 tends to antagonism for the linezolid-resistant isolates, providing another reason for the amplification of a linezolid-resistant population. Even though the rifampin dose was low at 200 mg daily, it produced sufficient selective pressure to allow resistant subpopulation amplification for this agent; the “α” value for the regimen for rifampin-resistant isolates was negative at −4.85.

We employed Monte Carlo simulation for the regimen of 600 mg daily of linezolid plus 600 mg daily of rifampin. We undertook two different approaches. In the first, we simply took the optimal cell kill and resistance suppression exposures and calculated how often a regimen of linezolid/rifampin of 600 mg/600 mg of the combination achieved those exposures. The results were clear cut. The optimal exposures for both were only achieved circa 58% of the time. Higher rifampin doses (20–30 mg/kg/day [25] increased the target attainment to 84–93%). Peloquin had first suggested earlier [26] that we were not administering optimal exposures to rifampin and these data are concordant with that suggestion.

We also employed the full mathematical model to perform a 1,000 subject simulation to calculate the total population burden, the fully susceptible population, as well as the burden of linezolid- and rifampin-resistant organisms over time. We again chose the 600 mg/600 mg (both daily) regimen for this evaluation. The results are displayed in Figure 3. The median ± SD of the different populations is displayed in Panels A and B. The first important issue is that when a specific drug regimen is administered to a population of patients, the results will vary significantly because of inter-patient variability in the handling of both agents in the combination. Figure 3A shows the range of impact of a 600 mg linezolid plus 600 mg of rifampin regimen administered daily on the total bacterial population.

Panel B displays delineation of the disparate effects on the different subpopulations. The combination regimen of 600 mg/600 mg of linezolid/rifampin drives first order decline in the fully-susceptible subpopulation. By experiment end, over 50% of simulated subjects have eradicated this subpopulation. For those with lower exposures of one drug, the other or both, there is some amplification of subpopulations resistant to linezolid or rifampin. In the last portion of the experiment, we are, for many simulated subjects, simply trading off fully susceptible organisms for resistant isolates.

Figure 3, panels C–F show the range of the impact on the total, fully susceptible and drug-resistant populations at experiment end. An eradication event (all populations) was achieved in 40/1000 iterates (4%). It should be noted that this outcome assumes the MIC is that of the isolate studied in these experiments. Resistant subpopulations were eradicated in slightly greater than 30% of instances for each drug in the combination.

The analysis demonstrates that additive combinations have an easier time having a major impact on the fully susceptible population relative to the resistant subpopulations. This is because the second drug is required to be high on the exposure-response curve in order to be able to suppress or kill the bacterial population less-susceptible to the other agent. The other factor to have an impact is the size of the change in MIC between the susceptible and resistant organisms. In this combination, rifampin has a major change in MIC (>32-fold increase). This means that the second drug (linezolid) is, in essence, acting alone on these organisms and suboptimal exposures straightforwardly lead to amplification of the population.

This may be modified if the two agents are highly synergistic instead of additive or antagonistic. Nonetheless, if we cannot find a synergistic pair, it becomes important to recognize that after a time the first combination regimen will have produced its maximal effect. It may then be wise to switch to a completely new regimen to help achieve an eradication event in a very high proportion of patients. The fully susceptible population will be eradicated in many subjects and much of the remaining organism burden will be resistant to one drug or another. A new regimen taking over at approximately one month will solve this problem if the new agents are independent of the resistance mechanisms affecting the first pair. In addition, because many patients will have a reduced bacterial burden because of the initial regimen, the probability of resistant subpopulation amplification will be reduced for the follow-on regimen.

It is important to emphasize that these findings are for Log-phase organisms. It is felt that there are other metabolic states of Mtb, such as slower growing organisms in acid environments as well as non-replicative persister phenotype organisms. Organisms also persist intracellularly. These other populations make the problem more complex. Nonetheless, as we learn more about the impact of combination therapy on these separate populations over time, the necessity to switch regimens during therapy becomes more important if we are to achieve the goal of markedly reducing the duration of therapy, while suppressing resistance.

We must emphasize that in order to kill optimally and suppress resistance amplification, it is not sufficient just to have a combination regimen. Preferably, the regimen should be synergistic or at least additive in all instances. Further, the doses chosen for each drug in the combination should be sufficient to suppress the amplification of the pre-existent, less-susceptible populations for each drug. The intensity of exposure that is optimal for each drug will be a function of the size of the change in MIC value between the wild-type isolate and the resistant mutant. As an example here, the MIC-value change for rifampin is so large that rifampin-resistant clones are being suppressed only by the second drug (linezolid in this instance).

It is critical to properly use the new drugs that are entering our therapeutic armamentarium. We have the opportunity to choose wisely so that we can achieve the dual goal of rapid bacterial kill and resistance amplification suppression. The mathematical model set forth here along with the Hollow Fiber Infection Model allows rational choice of combination regimens. Monte Carlo simulation allows between-patient variability in drug exposures to be accounted for and identify the rates of attainment of target exposures which will achieve the stated goals. Wise choices will prolong the therapeutic utility of new agents as well as allowing the greatest probability of identifying a regimen that will allow a shorter therapeutic duration.

## Materials and Methods

### Bacterium

*Mycobacterium tuberculosis* (*Mtb*) strain H37Rv was used. Stocks of the bacterium were stored at −80°C. For each experiment, an aliquot of the bacterial stock was inoculated into filter-capped T-flasks containing 7H9 Middlebrook broth that was supplemented with 0.05% Tween 80 and 10% oleic acid, albumen, dextrose and catalase (OADC). The culture was incubated at 37°C, 5% CO_2_ on a rocker platform for 4 to 5 days to achieve log phase growth.

Log-phase bacteria: Log phase growth bacteria were generated as described above. The bacteria were washed with fresh Middlebrook broth and were then transferred to pre-warmed 7H9 broth supplemented with 10% ADC (ADC-broth). The bacteria were adjusted to the desired concentration with pre-warmed ADC-broth and were inoculated into the hollow fiber cartridges. Log phase growth was maintained by continuously replacing the medium within the hollow fiber systems with fresh ADC-broth. Quantitative cultures of the starting inocula were conducted to confirm that the desired bacterial concentrations were placed into the hollow fiber systems. By serial dilution plating, quantitative estimations of the control bacterial cultures were conducted to confirm that bacteria were in log-phase throughout the course of the experiment.

### Drugs

Pharmaceutical grade linezolid was purchased as a solution for injection from CuraScript (St. Mary, FL). Rifampin powder was purchased from Sigma-Aldrich (St. Louis, MO). The drugs were stored according to the manufacturers' instructions.

Rifampin powder was dissolved in DMSO and then added to medium to the desired concentration. The working solutions of rifampin were stored at −80°C. Aliquots of the working solutions were thawed on the day of use and were used immediately. Linezolid solution for injection was dissolved with sterile water to the desired concentrations. The ADC-broth used in all arms of the hollow fiber experiments was supplemented with DMSO (final concentration: 0.3% DMSO). The growth of the *Mtb* in log phase was not affected by concentrations of DMSO as high as 1% (data not shown).

### Agar susceptibility testing

Susceptibility studies for linezolid and rifampin were conducted with log phase growth *Mtb* using the agar proportional method described by the CLSI (Susceptibility testing for Mycobacteria, Nocardiae, and Other Aerobic Actinomycetes; Approved Standard, Document A24-A, Wayne, PA) and the absolute serial dilution method on 7H10 agar +10% OADC and 0.3% DMSO. The MICs were read after 4 weeks of incubation at 37°C, 5% CO_2_. For the agar proportional method, the lowest concontration of a drug that provided a 99% reduction in the bacterial density relative to the no-drug control was read as the MIC. For the absolute serial dilution method, the MIC was read as the lowest concentration of drug for which there was no growth on the agar plate.

### Mutation frequency studies

Mutation frequencies were determined for 2.5x the MIC of linezolid and for the critical concentration of 1 µg/mL of rifampin. MICs to the test drug were determined for a subset of the mutants that were derived from the mutation frequency studies to define the change in MIC values in the drugs between the parent and mutant strains.

### Overview of the *In vitro* hollow fiber system

The methods for the HFIM for *Mycobacterium tuberculosis* study have been described elsewhere [9]. An *in vitro* hollow fiber system allows the investigator to expose a microbe to any concentration-time profile of antibiotics and can simulate the pharmacokinetic profile for any drug and any half-life within the system.

Syringe pumps infuse the drug(s) into the hollow fiber system at a rate to simulate an intravenous or oral route of administration of the compound(s) and the drug-containing medium is replaced with drug-free medium to simulate the desired half-life of the drug(s). Medium samples are taken from the central compartment for measurement of drug content by liquid chromatography dual mass spectrometry (LC/MS/MS) to confirm achievement of the targeted PK profile. The peripheral compartment is sampled for quantitative culture of the bacterium over the course of an experiment. Bacterial samples are plated on drug-free agar and agar supplemented with the antibiotic(s) infused into that hollow fiber system to characterize the effect of the treatment regimen on the total and less-susceptible bacterial populations.

Dose-range combination study for bolus dosed linezolid and rifampin against log phase growth *Mtb*. Different half-lives were developed in the system simultaneously employing the approach of Blaser [27]. Three dosages of linezolid and rifampin were administered alone and together as 2 h infusions on a once-daily schedule of administration. The simulated half-life for linezolid was 8.5 hours. The simulated half-life for rifampin was 3 hours.

### Population Combination Therapy Model

Population mixture modeling was performed employing the Non-Parametric Adaptive Grid (NPAG) program of Leary et al [28] and with an approach previously published by our laboratory [29]. Monte Carlo simulation was performed with the ADAPT V package of D'Argenio et al [30] and with PMetrics [31]. This approach allows us to apply classical properties of drug interaction for effect (Synergy, Additivity, Antagonism) to multiple populations simultaneously because of the mixture model approach. The original Greco model did not allow this and ignored the possibility of a resistant subpopulation. The first two differential equations are those required to describe the concentration-time profiles for each of the drugs in the combination. This requires 4 parameters, as the drug administration pumps will be set to describe a mono-exponential decline profile. The parameters are Volume (V in Liters) for Drug_1_ (V_1_) and Drug_2_ (V_2_) and Clearance for the 2 drugs, CL_1_ and CL_2_. These differential equations are displayed below:

(1) dX_1_/dT = R_1_ – (CL_1_/V_1_)×X_1_; where R_1_ is the piecewise input function for Drug_1_ and X_1_ is the Drug_1_ amount in the central compartment.

(2) dX_2_/dT = R_2_ – (CL_2_/V_2_)×X_2_; where R_2_ is the piecewise input function for Drug_2_ and X_2_ is the Drug_2_ amount in the central compartment.

The next two differential equations describe the growth and death of the drug-susceptible populations for Drug1 and Drug2. Differently from previous modeling we have done, the kill function will be the equation of the Universal Response Surface Approach (URSA) of Greco. This approach has been employed by our lab in the past for cell kill analysis. It is appropriate to employ this combination approach here, as the differential equation describes the growth (front part of the equation) and kill (back part) of the susceptible population.

(3) dN_S_/dT = _Kgmax-S_×N_S_×G – K_kmax-S_×M_S_×N_S_; where N_S_ is the number of organisms susceptible to Drug_1_ and Drug_2_, K_gmax-S_ is the maximal growth rate constant for the population sensitive to both Drug_1_ and Drug_2_, G is a logistic carrying function, which allows the population to achieve stationary phase, K_kmax-S_ is the maximal kill rate constant for Drug_1_ and Drug_2_ in combination for the susceptible population and M_S_ incorporates the URSA equation of Greco [18] for the Drug_1_ and Drug_2_-Susceptible population. Because the Greco equation is not in closed form, the parameters must be estimated via a bi-directional root finder. This has been implemented in the NPAG program, along with code to allow simultaneous handling of two agents by Van Guilder, Neely, Schumitzky and Jelliffe.

(4) dN_R1_/dT = K_gmax-R1_×N_R1_×G – K_kmax-R1_×M_R1_×N_R1_; where N_R1_ is the number of organisms resistant to Drug_1_ and sensitive to Drug_2_, K_gmax-R1_ is the maximal growth rate constant for the Drug_1_-resistant organisms, G is a logistic carrying function, which allows the population to achieve stationary phase, K_kmax-R1_ is the maximal kill rate constant for Drug_1_ and Drug_2_ in combination for the Drug_1_-resistant population and M_R1_ incorporates the URSA equation of Greco for the Drug_1_-resistant, Drug_2_-sensitive population.

(5) dNR_2_/dT = K_gmax-R2_×NR_2_×G – K_kmax-R2_×M_R2_×N_R2_; where N_R2_ is the number of organisms resistant to Drug_2_ and sensitive to Drug_1_, K_gmax-R2_ is the maximal growth rate constant for the Drug_2_-resistant organisms, G is a logistic carrying function, which allows the population to achieve stationary phase, K_kmax-R2_ is the maximal kill rate constant for Drug_1_ and Drug_2_ in combination for the Drug_2_-resistant population and M_R2_ incorporates the URSA equation of Greco for the Drug_2_-resistant, Drug_1_-sensitive population.

Normally, there would be a requirement for a sixth differential equation, describing the population resistant to both Drug_1_ and Drug_2_. However, we have not found such strains at baseline experimentally in our experiments. 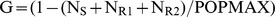


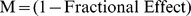
as derived from Greco URSA model; in this circumstance, E_con_ is set to 1.0.

For the Greco URSA model:
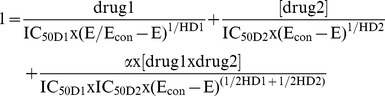
where [drug 1] is the concentration of Drug_1_; [drug 2] is the concentration of Drug_2_; IC_50D1_ is the concentration for which the effect is half maximal for Drug 1; IC_50D2_ is the concentration for which the effect is half maximal for Drug_2_; HD_1_ and HD_2_ are Hill's constants for Drug_1_ and Drug_2_, respectively; *E*_con_ is the effect for the control; α is the interaction parameter; and *E* is the fractional effect.

If α and its attendant 95% confidence bound cross zero, the effect is additive. If α and its attendant 95% confidence bound do not cross zero and are positive, the effect is synergistic. If α and its attendant 95% confidence bound do not cross zero and are negative, the effect is antagonistic.

The use of a mixture model allows independent identification of interaction parameters (α_1_ through α_3_) that identify the interaction of the drugs for the fully susceptible population (α_1_), as well as subpopulations resistant to Drug_1_ or Drug_2_ (α_2_ and α_3_). This will allow identification of regimens optimal for overall bacterial cell kill as well as resistance suppression for both agents.

System Outputs: System outputs 1 & 2, associated with differential equations 1 and 2 are the measured Drug_1_ and Drug_2_ concentrations in the central compartment 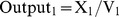; 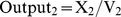.

System Output 3 is the Total Organism Number which is the Population sensitive to Drug_1_ and Drug_2_ plus population resistant to Drug_1_, sensitive to Drug_2_ plus population resistant to Drug_2_ and sensitive to Drug_1_ (as above, a population resistant to both Drug_1_ and Drug_2_ has not yet been observed). This output is measured by plating on antibiotic-free plates.

System Output 4 is the Population resistant to Drug_1_ and sensitive to Drug_2_. This output is measured by plating on agar into which Drug_1_ has been incorporated. The actual concentration employed will differ, depending upon the step size of the resistance mechanism that we are attempting to capture in any experiment with different drugs.

System Output 5 is the Population resistant to Drug_2_ and sensitive to Drug_1_. This output is measured by plating on agar into which Drug_2_ has been incorporated. The actual concentration employed will differ, depending upon the step size of the resistance mechanism that we are attempting to capture in any experiment with different drugs.

System Output 6 is the Population resistant to Drug_1_ and to Drug_2_. This output is measured by plating on agar into which Drug_1_ and Drug_2_ have been incorporated. The actual concentrations employed will differ, depending upon the step size of the resistance mechanism that we are attempting to capture. As above, we have not yet observed the need for this system output.

This approach to modeling combination chemotherapy with a mixture model and the URSA equation will allow the “inverted U” mountain type of response to be modeled as was demonstrated in our previous publication [32]. Because of the fully parametric nature of this approach, it allows Monte Carlo simulation to be conducted and allows powerful bridging to man. This approach allows us to explore combination chemotherapy for cell kill as well as resistance suppression for *Mycobacterium tuberculosis*, and also for any other circumstance requiring combination chemotherapy.
